# Continuous-Flow System for Methylene Blue Removal Using a Green and Cost-Effective Starch Single-Rod Column

**DOI:** 10.3390/polym15193989

**Published:** 2023-10-04

**Authors:** Tarawee Taweekarn, Worawit Wongniramaikul, Wilasinee Sriprom, Wadcharawadee Limsakul, Aree Choodum

**Affiliations:** Integrated Science and Technology Research Center, Faculty of Technology and Environment, Prince of Songkla University, Phuket Campus, Kathu, Phuket 83120, Thailand; tarawee.t@phuket.psu.ac.th (T.T.); worawit.won@phuket.psu.ac.th (W.W.); wilasinee.s@phuket.psu.ac.th (W.S.); wadcharawadee.n@phuket.psu.ac.th (W.L.)

**Keywords:** methylene blue, monolithic column, starch cryogel, continuous-flow adsorption, water treatment, adsorption

## Abstract

A continuous-flow system based on a green and cost-effective monolithic starch cryogel column was successfully developed for removing methylene blue (MB). The proposed column exhibited high removal efficiency (up to 99.9%) and adsorption capacity (25.4 mg·g^−1^) for synthetic and real samples with an adsorbent cost of USD 0.02. The influence of various operation parameters, including the flow rate, initial concentration, column height, and temperature, on the MB removal efficiency was examined and reported. The MB removal efficiency remained >99% in the presence of potential interferences, highlighting the good performance of the cryogel column. The Yoon–Nelson dynamic model explained the MB adsorption better than the Bohart–Adams model, as indicated by the higher R^2^ values (R^2^ = 0.9890–0.9999) exhibited by the former and current trends of its parameters. The MB removal efficiency of the cryogel column remained at 62.7% after three reuse cycles. The wastewater containing MB collected from a local batik-production community enterprise in Phuket, Thailand was applied to the proposed continuous-flow system under optimum conditions, and results indicated that 99.7% of the MB present in 2.4 L of wastewater was removed. These results validate the excellent application potential of the cryogel column for the continuous-flow adsorption of MB. This study will facilitate future industrial applications and process designs of the continuous-flow system.

## 1. Introduction

Water contamination with dyes is a global issue. In particular, the textile sector, where enormous quantities of dye effluents are released from the manufacturing process, is a major culprit. It has been reported that approximately 12–14% of the 7 × 105 tons of dyestuff produced annually is discarded into the environment [1]. This is extremely concerning because of their poor biodegradability, high toxicity, carcinogenicity, and mutagenicity [2]. Methylene blue (MB) is one of the most employed dyeing substances for coloring silk, wool, cotton, and paper [3]. The discharge of MB effluents into natural streams without adequate treatment limits solar-light penetration and restricts the photosynthetic activity of aquatic plants, causing numerous environmental issues [4,5]. Consequently, the effective removal of MB from wastewater to prevent environmental pollution requires urgent attention.

Adsorption is one of the most appropriate methods for MB removal. The procedure is simple, fast, and inexpensive, and the adsorbent materials are abundant and easily recyclable [3,6]. Various adsorbents have been employed for MB removal, such as magsorbents [7,8], carbon-based adsorbents [9], hydrogels [10], and starch-based adsorbents [8,11]. Recently, the application of eco-friendly and low-cost adsorbents for MB removal has been reported [12,13,14]. Taweekarn et al. [12] applied a green starch cryogel for MB adsorption and reported a high removal efficiency of 81.58 ± 0.59% and an adsorption capacity of 34.84 mg·g^−1^. The application of a renewable honeycomb-biomass adsorbent based on oxidized corn starch and gelatin extracted from leather solid wastes for MB removal has also been reported, affording an excellent adsorption capacity of 1551.5 mg·g^−1^ [13]. Additionally, the application of an eco-friendly, superabsorbent hydrogel film based on cassava starch and polyacrylic acid for MB adsorption has been reported, affording a high adsorption capacity of 1044 mg·g^−1^ and removal efficiency of 95% [14]. However, these studies mostly employed batch adsorption tests, which are useful for small-scale studies but not for practical applications [15]. As wastewater treatment plants commonly operate in a continuous system, studies conducted under continuous-flow conditions are necessary and preferred. In this regard, fixed-bed columns packed with a variety of adsorbents, such as cellulose nanocrystal–alginate hydrogel beads [16], water hyacinth–immobilized sodium alginate beads [17], untreated coffee residue [18], straw-based adsorbents [19], and Cu_2_O nanocomposite ceramsite [20], have been investigated for MB removal. Fixed-bed column systems provide various advantages, including high scalability for industrial applications and high pollutant removal efficiency from wastewater. Further, they can provide information on the pollutant concentration in the effluent versus time [21]. However, these systems have several drawbacks, including adsorbent attrition, feed channeling, and the nonuniform flow of adsorbent particles [21]. These problems can be overcome by employing monolithic adsorbents in continuous-flow adsorption systems [22,23]. Additionally, monolithic adsorbents display outstanding performance compared with traditional granular adsorbents because of their high surface area, porous nature, structure stability, and tunable functionality [24,25]. Recently, polyethyleneimine-based macroporous monolithic sponges were applied for the continuous-flow removal of MB, affording a higher dye-absorption efficiency than that obtained in batch adsorption experiments (amount = 16 mg, flow rate = 10 mL h^−1^, and inlet concentration = 300 mg L^−1^) [26].

In this work, a monolithic starch cryogel column was employed for the continuous-flow removal of MB to promote future automation and/or large-scale applications. The inexpensive starch cryogel was prepared by replacing the commonly used synthetic polymers (e.g., polyvinyl alcohol [24,25] and polyethyleneimine [26]) and toxic, expensive cross-linkers (e.g., glutaraldehyde) with starch and limewater, respectively. The effects of the flow rate, initial MB concentration, adsorbent height, temperature, and interferences on the column’s performance were evaluated. Theoretical break-through curve models, including the Adams–Bohart and Yoon–Nelson models, were applied to elucidate the column dynamics. Thereafter, the developed continuous-flow adsorption system was applied to remove MB from wastewater samples collected from a local batik-production community enterprise in Phuket, Thailand to assess its applicability. In addition, the reusability of the monolithic column was evaluated.

## 2. Materials and Methods

### 2.1. Materials

MB was acquired from Merck (Darmstadt, Germany), and 95% commercial-grade ethanol was provided by High Science Co., Ltd. (Songkhla, Thailand). Rice flour (Erawan Brand, Nakhon Pathom, Thailand), tapioca starch (Jaydee Brand, Nakhon Pathom, Thailand), and food-grade red lime were purchased from a local grocery store in Kathu (Phuket, Thailand). All standard solutions were prepared using ultrapure water from a water-purification system (Merck, Darmstadt, Germany).

### 2.2. Monolithic Column Preparation and Characterization

The monolithic starch cryogel column was prepared following the procedures in our previous works [12,27,28]. The gel precursor was prepared with the gelatinization of rice flour (12.5 g) and tapioca starch (3.75 g) in limewater (130 mL). The resulting solution (80 g) was transferred into a plastic syringe (volume: 50 mL, diameter: 3 cm, and length: 10 cm) and frozen overnight at −20 °C. Thereafter, the product was naturally thawed at room temperature for 3 h before undergoing three freeze–thaw cycles. The obtained monolithic cryogel was taken out of its container, cut into appropriate sizes (2.5, 5, and 7.5 cm in length), and returned into a plastic syringe prior to use.

Energy-dispersive X-ray spectroscopy (EDX) with field emission scanning electron microscopy (FESEM, FEI, Eindhoven, The Netherlands) was implemented to examine the topography and elemental composition of the cryogel before and after MB adsorption. The functional-group composition of the material was investigated using Fourier-transform infrared spectroscopy (FTIR) (Bruker, Bremen, Germany) with the KBr pellet method. X-ray diffraction (XRD) analysis was conducted using an X-ray diffractometer (Empyrean, PANalytical, Netherlands) with monochromatic Cu Kα radiation.

### 2.3. Continuous-Flow Adsorption and Models

**Figure 1:**
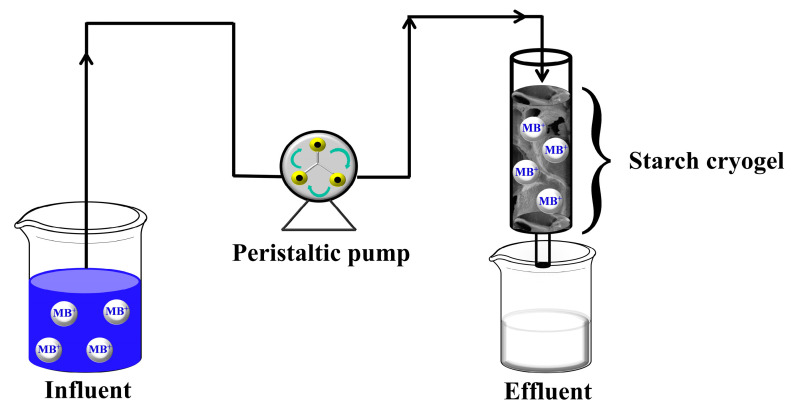
Layout of the continuous-flow system for MB adsorption with the starch cryogel monolithic column.

The in situ prepared cryogel monolithic column was installed in the continuous-flow system (Figure 1). Different lengths of the cryogel column (2.5, 5, and 7.5 cm) were employed for MB adsorption at various concentrations (C_i_ = 25–100 mg L^−1^) at a flow rate of 2.5–10 mL min^−1^, controlled with a peristaltic pump (Gilson, Middleton, WI, USA). The effluent samples were collected at regular intervals and analyzed for residual MB using a spectrophotometer until the MB concentration in the influent and effluent remained constant. Notably, the linear calibration curve used for the MB quantification at 664 nm (1–25 mg L^−1^, y = (0.11 ± 0.01)x + (0.22 ± 0.08), determination coefficient (R^2^) = 0.9912) was single-point recalibrated daily.

The break-through curve was established to demonstrate the performance of the cryogel column for continuous-flow MB removal. It was built by plotting the ratio of the MB concentration in the effluent at time t (C_t_) to the MB concentration in the influent (initial, C_i_) as a function of time at the appropriate column height. From this curve, the breakthrough time (t_b_) was determined to be C_t_/C_i_ = 0.1, i.e., when C_t_ reaches 10% of C_i_. The exhaustion time (t_e_) is the time at which C_t_ reaches 90% of C_i_, and it is defined as C_t_/C_i_ = 0.90 [16,29]. The effluent volume (V_eff_, mL) was calculated using Equation (1):

(1) $$V_{\mathrm{eff}}=Q\;t_{\mathrm{total}},$$

where Q is the volumetric flow rate (mL·min^−1^), and t_total_ (min) is the operation time when the adsorption column reaches saturation.

The empty bed contact time, which is the length of time an influent under treatment maintains contact with the monolithic column, is estimated using Equation (2):

(2) $$\mathrm{Empty}\;\mathrm{bed}\;\mathrm{contact}\;\mathrm{time}\;=\frac{V_{c}}{Q},$$

where V_c_ is the volume of the empty column (mL). The total MB adsorption capacity of the cryogel column (q_total_, mg) at the appropriate C_i_ and Q was estimated using Equation (3) [16,29,30]:

(3) $$\;Q_{\mathrm{total}}=\frac{Q}{100}\int_{t=0}^{t=t_{\mathrm{total}}}C_{\mathrm{ad}}dt\;=\frac{\mathrm{QA}}{1000}\;,$$

where C_ad_ is the adsorbed MB concentration (C_i_ − C_t_, mg·L^−1^), and A is the area above the break-through curve (C_t_/C_i_). The saturation loading capacity of MB on the cryogel monolithic column or the equilibrium MB uptake during continuous adsorption (q_e_ column, mg g^−1^) was calculated using Equation (4):

(4) $${\;q}_{e\;\mathrm{column}}=\frac{q_{\mathrm{total}}}{m_{\mathrm{cry}}},$$

where m_cry_ (g) is the dry mass of the cryogel material. The total amount of MB entering the column (m_total_, mg) was estimated using Equation (5):

(5) $$m_{\mathrm{total}}=\frac{Q.C_{i}.t_{\mathrm{total}}}{1000}.$$

The total percentage of adsorption (removal efficiency: %RE) related to the column performance was derived from the ratio of q_total_/m_total_ using Equation (6):

(6) $$\mathrm{Total}\;\mathrm{adsorption}\;(\mathrm{Removal}\;\mathrm{efficiency}:\;\%\mathrm{RE})\;(\%)=\left(\frac{q_{\mathrm{total}}}{m_{\mathrm{total}}}\right)\times 100.$$

The experimental data obtained from the continuous-flow adsorption experiment were fitted with kinetic and mass-transfer models, including the typically used Adams–Bohart and Yoon–Nelson models, to elucidate the dynamics of the MB adsorption on the cryogel monolithic column. The Adams–Bohart model assumes that the adsorption equilibrium is not instantaneous and, thus, that the adsorption rate is proportional to both the residual capacity of the adsorbent and the concentration of the adsorbed species [31,32]. It was employed for the initial part of the break-through curve (Ct/Ci = 0–0.5), where the adsorption is limited by the low mass transfer [31,33], as expressed in Equation (7):

(7) $$\ln\frac{C_{t}}{C_{i}}=k_{\mathrm{AB}}C_{i}t\;–\;k_{\mathrm{AB}}N_{0}\frac{Z}{F},$$

where k_AB_ (L·mg^−1^·min^−1^) is the Adams–Bohart constant rate (mass-transfer coefficient), N_0_ (mg·L^−1^) is the saturation concentration of the column, Z (cm) [34] is the height of the column, and F (cm·min^−1^) is the linear rate estimated by dividing the flow rate (mL·min^−1^) by the column area (cm^2^). The k_AB_ and N_0_ values were derived from the slope and intercept of the graph of $\ln\frac{C_{t}}{C_{i}}$ vs. t, respectively. The Yoon–Nelson model assumes that the rate of decrease in the adsorption probability for each adsorbate molecule is proportional to the probabilities of adsorbate adsorption and adsorbate breakthrough on the adsorbent [35,36]. Its linearized equation for a single-component system is expressed in Equation (8):

(8) $$\ln\frac{C_{t}}{C_{i\;}–C_{t}}=k_{\mathrm{YN}}t\;–\;\tau k_{\mathrm{YN}},$$

where k_YN_ is the model rate constant (min^−1^), and τ (min) is the time required for a 50% adsorbate breakthrough. The k_YN_ and τ values were derived from the slope and intercept of the ln (C_t_ ⁄ C_i_ − C_t_) vs. t graph, respectively.

### 2.4. Influence of Temperature

To study the effect of temperature on the MB removal efficiency of the cryogel monolithic column, continuous-flow adsorption studies were carried out under the following conditions: optimum initial concentration = 100 mg L^−1^, flow rate = 5 mL min^−1^, column length = 7.5 cm, and temperature = 25–45 °C.

### 2.5. Influence of Interference

The influence of interferences on the MB removal efficiency of the monolithic column was investigated. Sodium silicate (220 mg·L^−1^), metal ions (1 mg·L^−1^, Cu^2+^, Pb^2+^, Zn^2+^, K^+^, and Cd^2+^) [37,38], urea and sodium sulfate (1000 mg·L^−1^), and cationic crystal violet dye (100 mg·L^−1^) were mixed with an MB standard solution (100 mg·L^−1^) and applied to the continuous-flow system under optimum conditions. The removal efficiencies of MB in the presence of various interferences were compared with that obtained from the solution without interference.

### 2.6. Reusability of the Monolithic Cryogel

The desorption of the adsorbed MB on the monolithic cryogel was investigated using ethanol according to a previous report [39]. The MB-adsorbed cryogel column was removed from the container and soaked in ethanol (100 mL) for 5 min, after which the resultant solution was discarded. Thereafter, fresh ethanol was provided to prevent the re-adsorption of MB onto the adsorbent, and this serial desorption experiment was performed for 30 min. Thereafter, the column was immersed in 1 L of ethanol solution overnight, dried in an oven at 100 °C, and stored in zip-lock plastic bags for subsequent use. The used column was soaked in water for 20 min prior to use.

### 2.7. Real Sample Application

A real wastewater sample with a blue color was obtained from a local batik-production community enterprise in Phuket. It exhibited a maximum adsorption wavelength of 664 nm, corresponding to the MB standard solution at a concentration of 3.6 mg·L^−1^. The sample was pumped through a 7.5 cm high starch cryogel monolithic column at a flow rate of 5.0 mL·min^−1^ for the continuous-flow adsorption experiment. The effluent samples were collected at regular intervals and analyzed for residual MB using a spectrophotometer, as performed for the synthetic sample. Additionally, high-performance liquid chromatography was implemented for further confirmation.

## 3. Results and Discussion

### 3.1. Characterization of the Cryogel Monolithic Column

**Figure 2:**
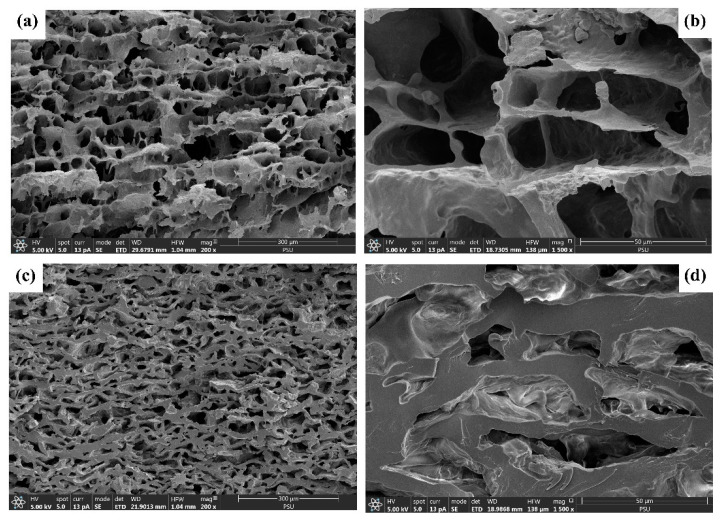
Field emission scanning electron micrographs of the monolithic starch cryogel before (**a**,**b**) and after (**c**,**d**) the continuous-flow adsorption of MB (**a**,**c** = 200×; **b**,**d** = 1500×).

The FESEM images of the monolithic columns before (Figure 2a,b) and after the equilibrium adsorption of MB reveal the presence of an interconnected network with macropores, which is a characteristic of the cryogel material [12,40,41,42]. Similar to the materials in the batch system, the surface homogeneity and smoothness of the materials increased after the equilibrium adsorption of MB (Figure 2c,d), suggesting the coating of dye molecules on the materials [12]. Furthermore, the wall thickness of the cryogel increased dramatically, reducing the flow through the pores. This may be attributed to the entrapping of MB and/or swelling of the material. These results are distinctly different from the results obtained for the batch system, attributed to the higher volume of water sample passing through the column in the continuous-flow system as well as the longer residence time, which promotes swelling.

**Figure 3:**
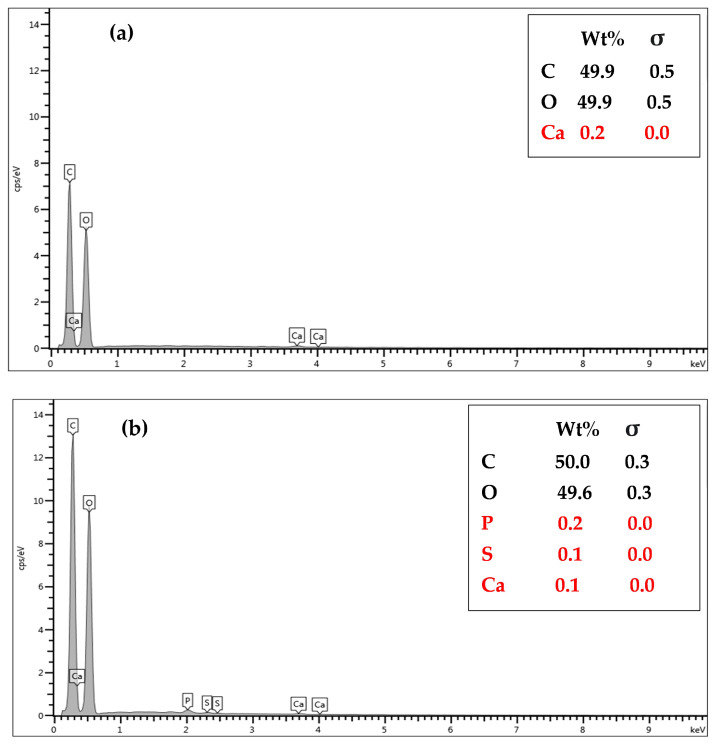
Energy-dispersive X-ray spectra of the monolithic starch cryogel before (**a**) and after (**b**) the continuous-flow adsorption of MB.

The EDX results for the continuous-flow system (Figure 3a,b) were similar to those reported for the batch system [12], with slightly lower calcium (0.2 to 0.1 wt%) and oxygen (49.9 to 49.6 wt%) contents observed for the former. In addition to the slight increase in the carbon content (49.9 to 50 wt%), sulfur, a constituent of the MB molecule, was detected (0.1 wt%), confirming the adsorption of MB on the cryogel column. The blue color of the column after the adsorption also served as a confirmation.

**Figure 4:**
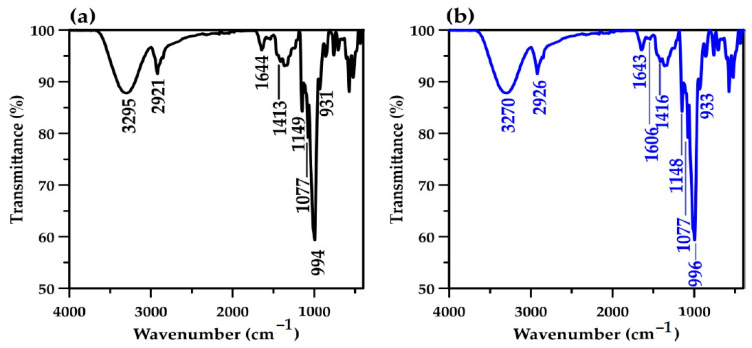
Fourier-transform infrared spectra of the monolithic starch cryogel before (**a**) and after (**b**) the continuous-flow adsorption of MB.

The FTIR spectra of the starch cryogel monolithic column exhibited characteristic peaks similar to those observed in previous works [12,40,41,42,43]. However, some differences were found when compared with those obtained for batch systems [12]. The most intense peaks of the prepared monolithic column before and after the continuous-flow adsorption were observed at 1077–931 cm^−1^ (Figure 4a,b), attributed to the amylopectin C–O bonding in the starch molecules [44]. This contrasts the high-intensity O–H stretching peak at 3443 cm^−1^ reported for the batch system. In addition, the absorption band corresponding to the O–H stretching in the starch molecules was observed at 3295 cm^−1^ before adsorption, instead of at 3443 cm^−1^ [12], and it presented at 3270 cm^−1^ after adsorption. The high intensity of the peak at 1077 cm^−1^ for the cryogel after MB adsorption (Figure 4b) may be due to the overlap of the C–S–C vibrations in the MB molecules [45]. In Figure 4b, the peak at 1606 cm^−1^ corresponding to CH=N from the MB molecules [45] indicates the adsorption of MB on the monolithic column. The band corresponding to the C–H stretching of starch molecules slightly shifts from 2921 cm^−1^ to 2926 cm^−1^ after MB adsorption, overlapping with the band at 2924 cm^−1^ and corresponding to the C–H bond in the MB molecules [46]. The absorption band attributed to the H–O–H bending in water molecules and/or C–O bending in amylopectin [12,26,41] remained at ~1644 cm^−1^, similar to the results for the batch system. The results from this continuous-flow system confirmed that the MB adsorption is attributable to physical interactions, considering the slight variation in the wavenumber (<10 cm^−1^) [47].

**Figure 5:**
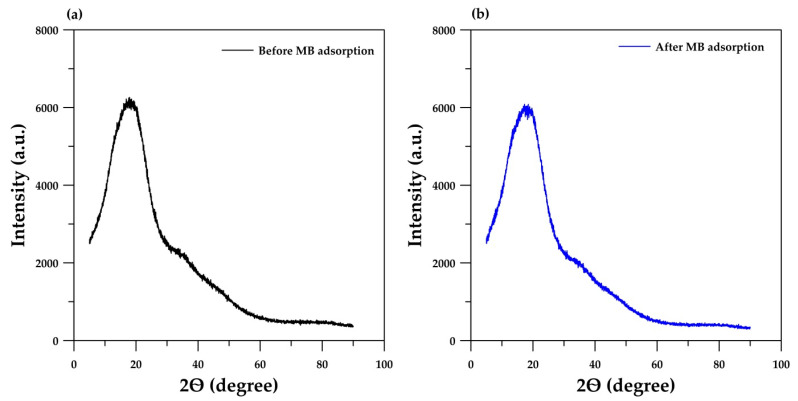
XRD patterns of the monolithic cryogel before (**a**) and after (**b**) the continuous-flow adsorption of MB.

The XRD patterns of the monolithic cryogel before and after the adsorption of MB are presented in Figure 5. The patterns before and after the adsorption are similar, with a large hump centered at 2θ = 20°. This indicates that the cryogel retains its amorphous structure [27,28] after adsorption.

### 3.2. Influence of Continuous-Flow Parameters

The effects of parameters, including C_i_, flow rate, column height, temperature, and interferences, on the continuous-flow system’s performance are discussed.

#### 3.2.1. Initial MB Concentration

**Figure 6:**
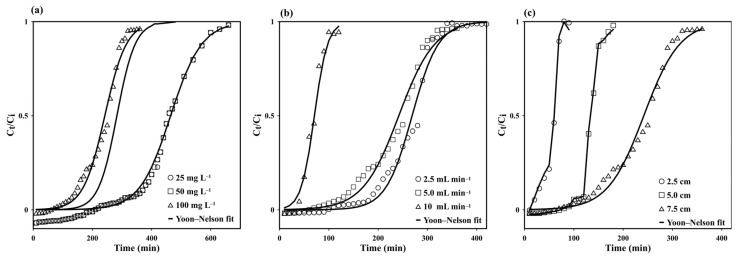
Effects of the (**a**) initial MB concentration in the influent solution, (**b**) flow rate, and (**c**) column height on the break-through curve of MB adsorption on starch cryogel, along with the break-through curves obtained using the Yoon–Nelson model.

The initial dye concentration in the inlet flow is one of the main variables and limiting factors of continuous-flow systems [43] because a given mass of adsorbent material can only adsorb a fixed amount of dye. The higher the dye concentration in the influent, the lower the influent volume that a fixed mass of adsorbent (the cryogel, in this case) can purify [35]. In this experiment, C_i_ was varied between 25 and 100 mg L^−1^ (at pH ~5.1, without pH adjustment), with a flow rate of 5 mL min^−1^ and a column length of 7.5 cm. Figure 6a displays the resulting break-through curves, and Table 1 summarizes the calculated parameters. At low C_i_ values, more time is required for the available active sites in the cryogel to reach full saturation. This is due to the relatively low diffusion coefficient, resulting from the low adsorbate dose and the slow transport of MB molecules. The slow transport is due to the low concentration gradient of MB ions on the adsorbent and in the bulk fluid [16,35]. The break-through curve at the low C_i_ was comparatively flat, indicating the formation of a relatively large mass-transfer zone at the column front [43] where MB adsorption occurs. At high C_i_ values, sharp break-through curves can be observed, suggesting the formation of a relatively small mass-transfer zone. Moreover, the break-through and exhaustion times decreased as C_i_ increased because of the corresponding coverage of more adsorption sites.

The adsorption capacity increased with C_i_ from 11.2 to 23.9 mg·g^−1^, attributed to the increased driving force (i.e., the high concentration gradient), which was valuable for overcoming the mass-transfer resistance [35,48]. However, the removal efficiency decreased from 98.2% to 96.4%. This occurred because, at low C_i_ values, all the MB molecules in the solution interact with the available binding sites of the cryogel, achieving partial saturation and increasing the adsorption efficiency. Conversely, at high C_i_ values, more MB molecules remain in the solution because of the full saturation of the binding sites, which decreases the adsorption efficiency [35,43,48]. Note that the removal efficiency obtained in the continuous-flow system is higher than that obtained in the batch system at the same C_i_ [12]. This may be attributed to the layout of the continuous-flow system, where the MB solution is pumped through the macroporous network of the column, increasing the possibility for MB molecules to contact and bind to the active sites of the cryogel. This contrasts the layout of the batch system, where the cryogel tablet is randomly shaken, and the MB contacts and binds to only the active sites on the column surface, despite the increased contact time.

#### 3.2.2. Flow Rate

The flow rate significantly influences the performance of the column in a continuous-flow system. Its effect on the adsorption of 100 mg·L^−1^ MB on a 7.5 cm high monolithic starch cryogel column was investigated in the range of 2.5–10 mL·min^−1^. The sorption capacity increased with the flow rate of the MB solution from 2.5 to 5 mL·min^−1^ (from 14.4 to 23.9 mg·g^−1^), whereas it decreased at 10 mL·min^−1^ (15.7 mg·g^−1^, Table 1). This was attributed to the reduced contact time at high flow rates and the strong competition among the numerous adsorbate molecules for the limited active sites on the adsorbent. Figure 6b shows the break-through curves at different flow rates. The adsorption was initially rapid, probably due to the availability of the active sites on the cryogel. Thereafter, the absorption rate decreased as the sites became increasingly occupied. The cryogel could accumulate MB even after the breakthrough, although with reduced efficiency. The steepness of the break-through curve increased with the flow rate, due to the reduced contact time, consequently reducing the removal efficiency. Moreover, the mass-transfer rate increased with the flow rate, increasing the adsorption efficiency of MB on the cryogel and, consequently, the saturation rate [35].

#### 3.2.3. Cryogel Column Height

Since the height of the monolithic cryogel column is correlated with the number of active sites that can accommodate MB molecules, it determines the adsorption capacity and removal efficiency, which are expressed by the steepness of the break-through curve. Here, the cryogel column height was varied between 2.5 and 7.5 cm while keeping C_i_ and the flow rate fixed at 100 mg·L^−1^ and 5 mL·min^−1^, respectively. The steepness of the break-through curve exhibits a strong dependence on the column height, as shown in Figure 6c. Increasing the column height from 2.5 to 7.5 cm led to an extension of the break-through point from 20 to 120 min and the exhaustion time from 70 to 300 min, due to the increased number of active sites on the cryogel [31]. The adsorption capacity and removal efficiency were also correspondingly enhanced (from 19.9 to 23.9 mg·g^−1^ and 94.4% to 96.4%, respectively) (Table 1) because more binding sites were available for the MB ions [49].

Furthermore, when the column height was increased, the axial dispersion in the mass transfer decreased, promoting the MB diffusion into the cryogel, and providing sufficient residence time for the interaction between the MB molecules and the deep binding sites in the cryogel column [35,48].

#### 3.2.4. Temperature

The effects of temperature on the MB adsorption capacity and removal efficiency of the monolithic cryogel were investigated at temperatures ranging from 25 °C to 45 °C. The adsorption capacity and the removal efficiency increased slightly from 23.9 to 25.3 mg g^−1^ and 96.40% to 99.67%, respectively, with an increase in temperature (Table 2). This is attributable to the decrease in the solution viscosity as the temperature increased, enhancing the mobility and diffusion rate of dyes across the external boundary layer and in the internal pores of the monolithic cryogel [50,51]. Additionally, the chemical interaction between the adsorbate and the surface functionalities of the adsorbent can be enhanced by increasing the temperature [51].

#### 3.2.5. Interferences

The interferences commonly present in batik wastewater include sodium silicate, metal ions (Cu^2+^, Pb^2+^, Zn^2+^, K^+^, and Cd^2+^) [37,38], the chemicals used in the batik process (urea and sodium sulfate), and cationic crystal violet dye. These materials were mixed with MB, and the resulting solution was applied to the continuous-flow system. The results showed that the removal efficiency of MB remained higher than 99% even with the interferences (Table 3), highlighting the excellent performance of the cryogel column. In addition, the crystal violet dye could be removed while maintaining the MB removal efficiency. These results validate the excellent performance of the monolithic column for MB removal even with complex matrices.

### 3.3. Application of Dynamic Models

#### 3.3.1. Application of the Adams–Bohart Model

The Adams–Bohart model was applied to the experimental data obtained from the continuous-flow experiment. The saturation sorption capacity (N_0_) of the monolithic cryogel column for MB was increased along with both the C_i_ (from 1906 to 4175 mg L^−1^ between 25 and 100 mg L^−1^, respectively) and the solution flow rate (from 2402 to 4790 mg L^−1^ between 2.5 and 10 mL min^−1^, respectively) (Table 4). When C_i_ was increased, k_AB_ decreased. This is in contrast with the results from the flow-rate influence study, where the model rate constant (k_AB_) increased with C_i_; therefore, this indicates that the overall system kinetics was dominated by external mass transfer in the initial stage of sorption in the cryogel column, similar to the adsorption mechanism using other adsorbent materials [48,52,53]. Although this model is a simple approach for evaluating the column dynamics, its validity is limited to the initial part of the break-through curve. Thus, the poor correlation coefficient reflects its poor applicability, which is consistent with other reports [49,52,53].

#### 3.3.2. Application of the Yoon–Nelson Model

The Yoon–Nelson parameters obtained for the MB adsorption on the monolithic cryogel column are summarized in Table 4. On increasing the column height from 2.5 to 7.5 cm, the time required for a 50% adsorbate breakthrough (τ) increased from 51 to 233 min, whereas the model rate constant (k_YN_) decreased from 0.119 to 0.028 min^−1^. Moreover, τ decreased when C_i_ was increased, similar to the results obtained with other adsorbent materials, such as chitosan/clay composites [48] and Garcinia-mangostana-peel-based granular activated carbon [52]. For the linearized model, the R^2^ values ranged between 0.9654 and 0.9695 for C_i_, 0.9393 and 0.9654 for the solution flow rate, and 0.9265 and 0.9753 for the column height. However, the nonlinear equation [47] showed better fitting than the linearized one, affording higher R^2^ values (0.9890–0.9999) with low standard deviation (line in Figure 6a–c). This result indicates that the Yoon–Nelson model is appropriate for predicting the adsorption dynamics of MB on the proposed monolithic starch cryogel column.

### 3.4. Reusability of the Monolithic Cryogel

The reusability of the monolithic cryogel column was investigated via the desorption of the adsorbed MB on the column using ethanol, according to the previous report [39]. The MB-adsorbed cryogel column was easily removed from the container as a whole piece without any filtration. The sharpness of the break-through curves increased following regeneration, indicating the presence of a relatively small mass-transfer zone. As shown in Table 5, as the number of catalyst-reuse cycles increased, the breakthrough and exhaustion times decreased. This is because, with each reuse, fewer adsorption sites become available for adsorbate uptake as some of them are covered and could not be efficiently desorbed in the previous cycle [54]. The removal efficiency decreased from 99.8% to 62.7% after three cycles. Since the monolithic cryogel column costs only THB 0.71 (USD 0.02), the cost per cycle is only USD 0.007. The good reusability of the column highlights its sustainability and environmental friendliness since less materials and energy are required to produce a new removal column for subsequent treatment. Additionally, since the monolithic column is prepared from a natural, biodegradable polymer (starch), instead of the commonly used synthetic polymers, the materials and energy expended for its production, as well as the generated wastes, would be less detrimental to the environment, potentially mitigating the microplastic problem. Moreover, the MB recovered from the spent column can be employed for other applications, in line with the concept of a circular and green economy for sustainability.

### 3.5. Real Sample Application

**Figure 7:**
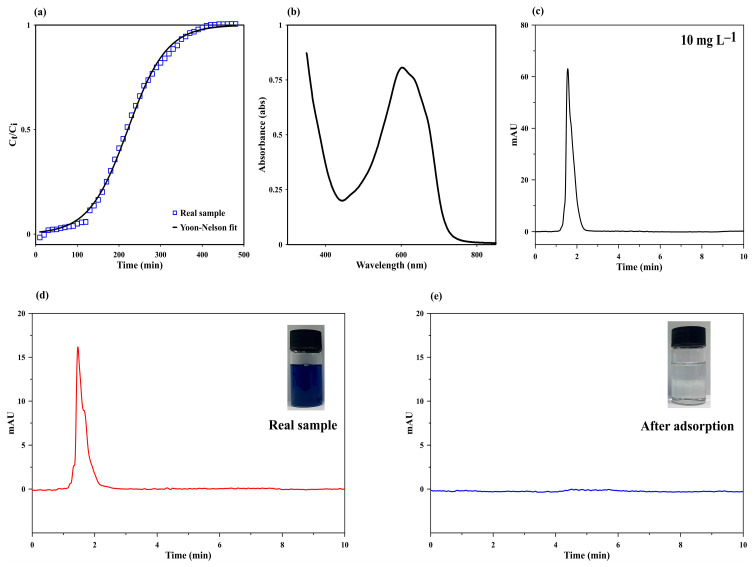
(**a**) The break-through curve of MB in wastewater adsorption using starch cryogel, along with the break-through curves obtained using the Yoon–Nelson model. (**b**) Spectrum of the wastewater analyzed using a spectrophotometer. (**c**) Chromatogram of the MB standard at 10 mg L^−1^ of MB in the wastewater sample (**d**) before and (**e**) after adsorption, analyzed using high-performance liquid chromatography-diode array detection (HPLC–DAD).

The monolithic cryogel was applied for continuous MB removal in wastewater collected from a local batik-production community enterprise in Phuket, Thailand. The wastewater, taken from the second rinsing step, contained 3.6 mg L^−1^ of MB, with pH 6.05, salinity < 1% (based on the detection limit of the salinity meter used), total suspended solid (TSS) = 22.7 ± 2.3 mg L^−1^, total dissolved solid (TDS) = 185.3 ± 6.9 mg L^−1^, and chemical oxygen demand (COD) = 158.4 mg L^−1^. After continuous removal using the monolithic starch cryogel, 99.7% of the MB present in the 2.4 L of wastewater was removed (Table 5). The break-through curve for the wastewater (Figure 7a) fitted well with the Yoon–Nelson model. The spectrum of the real wastewater sample obtained using a spectrophotometer is shown in Figure 7b, and the chromatograms of the influent and effluent from the continuous-flow system are shown in Figure 7d,e, respectively. The effluent volume (V_eff_) from the real sample (2400 mL) is equal to that from the synthetic water with an initial MB concentration of 50 mg L^−1^ (see Table 1), although the MB concentration in the real sample is considerably less than that in the synthetic water. The q_total_ in the real sample (8.57 mg) was also less than that in the synthetic water (117.7 mg). This may be attributed to the complexity of the pollutants present in the wastewater from the batik process [37,38]. In the batik process, there are three major sequential steps (soaking, boiling, and rinsing), each featuring unique and shared contaminants. These steps increase the complexity of wastewater treatment. However, the main pollutants, including waxes, dyes, silicate, and heavy metals, are present in all steps at different concentrations [37]. In the wastewater taken after the second rinse, the presence of various metal ions has been reported at concentrations of less than 1 mg L^−1^, except for calcium (4.32 mg L^−1^) and silica (49.95 mg L^−1^) [37]. However, Section 3.2.5 conveys that the removal efficiency of MB remained >99% when MB was mixed with sodium silicate (220 mg L^−1^, equivalent to 50 mg of L^−1^ silica) as well as metal ions (1 mg L^−1^). Thus, the decrease in the MB removal efficiency of the starch column from real samples may be unrelated to the presence of sodium silicate and metal ions. Therefore, it appears that contaminated wax may contribute to the reduction in the q_total_ of MB in the real sample. Since TSS, TDS, and COD after treatment were reduced to 13.33 ± 2.3 mg L^−1^, 116.0 ± 8.3 mg L^−1^, and 70.4 mg L^−1^, respectively, contaminated wax may also be removed using the starch cryogel, accounting for the reduction in the q_total_ of MB.

The performance of the monolithic starch cryogel column for the continuous-flow adsorption of MB was comparable to those of other fixed-bed materials, as shown in Table 6. The adsorption capacity of the starch monolithic cryogel was higher than that of the hydrogel bed [16,17]. The results highlight the application potential of the proposed cryogel for the treatment of real samples, with high removal efficiencies and good reusability.

## 4. Conclusions

Monolithic starch cryogel was demonstrated to be a green and cost-effective column for continuous-flow MB removal, with a high removal efficiency and adsorption capacity. The influence of various operation parameters, including the flow rate, initial concentration, column height, and temperature, on the MB removal efficiency was examined. The breakthrough time decreased as the flow rate increased because MB had less contact with the starch cryogel, lowering the removal efficiency of MB. The removal efficiency and the breakthrough time of MB decreased as the initial MB concentration increased. The breakthrough time and removal efficiency increased with the height of the cryogel column as well as the temperature. The removal efficiency of MB remained higher than 99% in the presence of potential interferences, highlighting the good performance of the cryogel column. In addition, crystal violet dye could be removed while maintaining the removal efficiency for MB. The Yoon–Nelson dynamic model better explained the MB adsorption than the Bohart–Adams model, as evidenced by the higher R^2^ values and the trend of the model’s parameters. The removal efficiency of the starch cryogel remained at 62.7% after three reuse cycles. The wastewater containing MB collected from the local batik-production community enterprise in Phuket, Thailand was applied to the proposed continuous-flow system under optimum conditions, and 99.7% of the MB present in 2.4 L of wastewater was removed. However, the q_total_ of MB in the real sample was less than that in the synthetic water, due to the complexity of pollutants present in the wastewater from the batik process. These results validate the excellent application potential of starch cryogel for the continuous-flow adsorption of MB. This study will facilitate future industrial applications and process designs of the continuous-flow system.

## Figures and Tables

**Table 1:** Parameters derived from the break-through curves under various conditions.

Flow Rate (mL·min^−1^)	H (cm)	C_0_ (mg·L^−1^)	T_b_ (min)	t_total_ (min)	V_eff_ (mL)	m_total_ (mg)	q_total_ (mg)	q_e_ (mg·g^−1^)	RE ^a^ (%)	EBCT ^b^ (min)
5.0	7.5	25	370	660	3300	82.5	81.0	11.2	98.2	7.36
5.0	7.5	50	240	480	2400	120	117.7	16.2	98.0	7.36
5.0	7.5	100	150	360	1800	180	173.5	23.9	96.4	7.36
2.5	7.5	100	200	420	1050	105	103.6	14.4	98.7	14.72
5.0	7.5	100	150	360	1800	180	173.5	23.9	96.4	7.36
10.0	7.5	100	50	120	1200	120	113.6	15.7	94.7	3.68
5.0	2.5	100	30	90	450	45	42.5	18.9	94.4	2.45
5.0	5.0	100	120	180	900	90	86.7	19.4	96.4	4.91
5.0	7.5	100	150	360	1800	180	173.5	23.9	96.4	7.36

^a^ Removal efficiency; ^b^ empty bed contact time.

**Table 2:** Parameters derived from the break-through curves at various temperatures.

Temperature (°C)	T_b_ (min)	T_total_ (min)	V_eff_ (mL)	q_total_ (mg)	m_total_ (mg)	q_e_ (mg g^−1^)	RE (%)
25	150	360	1800	173.50	180	23.90	96.40
35	210	360	1800	179.41	180	25.30	99.67
45	260	360	1800	179.52	180	25.32	99.74

**Table 3:** Parameters derived from the break-through curves with various interferences.

Samples		Parameters					
	T_b_ (min)	T_total_ (min)	V_eff_ (mL)	q_total_ (mg)	m_total_ (mg)	q_e_ (mg g^−1^)	RE (%)
MB (100 mg L^−1^)	150	360	1800	179.6	180	24.9	99.8
MB + CV ^a^	160	360	1800	179.9	180	25.4	99.9
MB + metal ions ^b^	250	360	1800	179.8	180	25.2	99.9
MB + urea + sodium sulphate ^c^	210	360	1800	179.8	180	25.1	99.9
MB + sodium silicate ^d^	170	360	1800	178.21	180	24.6	99.0

^a^ 100 mg L^−1^ of crystal violet; ^b^ 1 mg L^−1^ of Cu^2+^, Pb^2+^, Zn^2+^, K^+^, and Cd^2+^; ^c^ 1000 mg L^−1^ of urea and sodium sulphate; and ^d^ 220 mg L^−1^ of sodium silicate.

**Table 4:** Parameters derived from applying the Adams–Bohart and Yoon–Nelson models to the continuous-flow data.

Parameters	Adams–Bohart Model			Yoon–Nelson		
	K_AB_ × 10^−4^ (L·mg^−1^·min^−1^)	N_0_ (mg·L^−1^)	R^2^	K_YN_ (min^−1^)	τ (min)	R^2^
Initial conc. (mg L^−1^)						
25	4.76	1906	0.8635	0.0191	464.22	0.9695
50	3.30	3825	0.9265	0.0247	290.98	0.9670
100	1.66	4175	0.8502	0.0282	233.35	0.9654
Flow rate (mL min^−1^)						
2.5	1.61	2402	0.8652	0.0333	257.62	0.9564
5.0	1.66	4175	0.8502	0.0282	233.35	0.9654
10.0	3.23	4790	0.7525	0.0747	70.68	0.9393
Column height (cm)						
2.5	4.42	3318	0.9514	0.1194	51.24	0.9265
5.0	5.13	3336	0.9003	0.0899	136.34	0.9753
7.5	1.66	4175	0.8502	0.0282	233.35	0.9654
Real sample	1.79	196.6	0.6338	0.0283	262.44	0.9604

**Table 5:** Parameters derived from the break-through curves for the reusability experiment and real sample application.

Time of Use/Sample	Flow Rate (mL min^−1^)	H (cm)	C_0_ (mg L^−1^)	T_b_ (min)	t_total_ (min)	V_eff_ (mL)	m_total_ (mg)	q_total_ (mg)	q_e_ (mg g^−1^)	RE ^a^ (%)	EBCT ^b^ (min)
1	5.0	7.5	100	150	360	1800	180	179.6	24.9	99.8	7.36
2	5.0	7.5	100	30	180	900	90	80.2	8.7	89.1	7.36
3	5.0	7.5	100	10	90	450	45	28.2	2.7	62.7	7.36
RW ^c^	5.0	7.5	3.6	200	480	2400	8.59	8.57	1.19	99.7	7.36

^a^ Removal efficiency; ^b^ empty bed contact time; and ^c^ real wastewater sample.

**Table 6:** Removal of MB via continuous-flow adsorption using various materials.

Adsorbent	Initial Concentration (mg·L^−1^)	Flow Rate (mL·min^−1^)	Bed Height (cm)	q_total_ (mg)	Reusability (Cycles)	Real Sample	Ref.
Cellulose nanocrystal–alginate hydrogel beads	250	4.17	7.4	19.37	5	-	[16]
Alginate–water hyacinth beads	20	1.5	2.5	-	3	-	[17]
Pine cone	70	12	10	147	-	-	[30]
Chitosan–clay composite	50	5.0	3.6	58.395	-	-	[48]
Monolithic cryogel based on starch	100	5.0	7.5	179.6	3	✓	This work

## Data Availability

All data are available from the corresponding author upon reasonable request.
